# Plasma calprotectin is extremely high in patients with lysinuric protein intolerance

**DOI:** 10.1002/jmd2.12377

**Published:** 2023-06-20

**Authors:** Mari Kärki, Laura Tanner, Satu Lahtinen, Tero Soukka, Harri Niinikoski

**Affiliations:** ^1^ Department of Pediatrics University of Turku Turku Finland; ^2^ Department of Clinical Genetics Helsinki University Hospital Helsinki Finland; ^3^ Department of Medical and Clinical Genetics University of Helsinki Helsinki Finland; ^4^ Department of Life Technologies/Biotechnology University of Turku Turku Finland; ^5^ Institute of Biomedicine University of Turku Turku Finland

**Keywords:** Finnish disease heritage, hypercalprotectinemia, Lysinuric protein intolerance, renal insufficiency

## Abstract

Lysinuric protein intolerance (LPI) is a rare autosomal recessive disorder affecting the transport of cationic amino acids. Elevated plasma zinc concentrations have been described in patients with LPI. Calprotectin is a calcium‐ and zinc‐binding protein, produced by polymorphonuclear leukocytes and monocytes. Both zinc and calprotectin have an important role in immune system. In this study, we describe plasma zinc and plasma calprotectin concentrations in Finnish LPI patients. Plasma calprotectin concentration was measured from 10 LPI patients using an enzyme‐linked immunosorbent assay (ELISA) and it was remarkably high in all LPI patients (median: 622 338 μg/L) compared to that in healthy controls (608 μg/L). Plasma zinc concentration was measured by photometry and it was normal or only mildly elevated (median: 14.9 μmol/L). All the patients had decreased glomerular infiltration rate (median: 50 mL/min/1.73 m^2^). In conclusion, we observed extremely high plasma calprotectin concentration in patients with LPI. Mechanism of this phenomenon is unknown.

**Synopsis**

Plasma calprotectin is extremely high in patients with LPI.

## 1 INTRODUCTION

Lysinuric protein intolerance (LPI) is a rare inherited autosomal recessive disorder of amino acid metabolism, which effects the transport of dibasic cationic amino acids lysine, arginine, and ornithine. LPI has been described worldwide but it is more prevalent in Finland (1/60 000) than in any other country.^1^, ^2^ LPI is caused by mutations in the *SLC7A7* gene (solute carrier family 7, member 7) encoding y + LAT‐1 protein, the catalytic light chain subunit of the heteromeric amino acid transporter located at the basolateral membrane of the epithelial cells of the renal proximal tubules and intestine. All Finnish patients share the same homozygous variant, c.895‐2A > T (NM_001126105.2).^3^, ^4^, ^5^ The loss of function of the transporter leads to reduced intestinal absorption of lysine, arginine, and ornithine and their increased excretion in the urine, causing depletion of these amino acids in the blood. Deficiency of arginine and ornithine leads to impaired urea cycle function, resulting in protein aversion and hyperammonemia after dietary protein loads. Majority of the patients develop protective aversion to protein‐rich foods at an early age. LPI is a complex multisystem disease and the spectrum of symptoms is wide, varying from nearly normal growth to severe multi‐organ disease. The principal symptoms of LPI include failure to thrive, growth retardation, muscular weakness, osteoporosis, hepatosplenomegaly, combined hyperlipidemia, and hematological and immunological defects.^6^, ^7^, ^8^, ^9^ Renal insufficiency is a common complication that may progress to end‐stage renal disease.^10^ Life‐threatening pulmonary complications, including pulmonary fibrosis and alveolar proteinosis, may occur.^11^, ^12^ The treatment is based on dietary protein restriction and supplementation with oral L‐citrulline.^2^

Due to the protein‐restricted diet, patients with LPI are at risk for nutritional deficiencies. Because meat and seafood are considered major dietary sources of zinc,^13^ it has been suspected that LPI patients might suffer from zinc deficiency. Daily dietary intake of zinc in adult patients with LPI has been between 8.0 and 8.3 mg per day,^14^ that is rather close to the population reference intake of 7.5 mg in females and 9.4 mg in males.^15^ However, high plasma zinc concentrations have been observed in many Finnish LPI patients (unpublished observation; Table 2), but his phenomenon has not been systematically studied.

Zinc is an essential trace element, occurring as a component at least 300 enzymes and participating in numerous cellular functions.^16^ Only 0.1% of total body zinc is located in the plasma. Most of the plasma zinc is bound to proteins, mainly albumin (80%–85%).^17^, ^18^ Calprotectin (MRP8/14, S100A8/A9), a heterodimer of S100A8 and S100A9, is a calcium‐ and zinc‐binding protein, produced mainly by polymorphonuclear leukocytes and monocytes.^19^, ^20^ It has various biological functions, including antimicrobial, apoptosis‐inducing and chemotactic activities.^20^, ^21^ Elevated calprotectin levels in plasma or blood have been detected in several inflammatory diseases, such as inflammatory bowel diseases, rheumatoid arthritis and cystic fibrosis.^22^, ^23^, ^24^ Calprotectin plays an important role in nutritional immunity by chelating essential nutrients zinc and manganese and creating zinc‐limited microenvironments, leading to bacterial metal starvation.^25^, ^26^ Furthermore, zinc deficiency has been linked to the upregulation of calprotectin.^27^, ^28^

Plasma calprotectin levels in LPI patients have not been reported before. In this study, we describe in detail plasma calprotectin and plasma zinc concentrations of 10 Finnish LPI patients.

## 2 PATIENTS AND METHODS

10 Finnish LPI patients (six female) followed‐up at the Turku University Hospital were included in this study. We included only adult patients with genetically confirmed LPI, otherwise there were no specific inclusion or exclusion criteria. The median age of the patients was 46.5 years (range 27–65 years). All patients followed a protein‐restricted diet instructed by a nutritionist. Daily use of a multivitamin preparation containing 10 mg of zinc was recommended for all patients. Otherwise, no zinc supplementation was included in their treatment protocol. All patients were clinically examined, and none of the patients had signs of acute infections. None of the patients were on anti‐inflammatory medication. All patients used oral L‐citrulline. Eight patients were treated with cholesterol‐lowering drugs (statins). Six patients were treated with antihypertensive drugs (ACE inhibitors or AT2‐blockers). Eight patients had lysine supplementation. Two patients needed oral supplementation of sodium bicarbonate and six patients used sodium benzoate.

Routine follow‐up laboratory tests were analyzed, including total blood cell count, plasma creatinine, serum cystatin C, urine beta‐2 microglobulin, plasma alanine aminotransferase (ALT), plasma alkaline phosphatase (ALP), plasma ammonium ion (NH_4_), plasma iron, plasma zinc, plasma copper, blood manganese, plasma calcium, total plasma cholesterol, high‐density lipoprotein (LDL), triglycerides, prealbumin, plasma, and urinary amino acids. Glomerular filtration rate (GFR) was calculated using the Chronic Kidney Disease Epidemiology Collaboration equation (CKD‐EPI). All laboratory analyses were performed using standard clinical laboratory methods. Ferritin and LDH levels were not routinely measured as they are constantly elevated in LPI patients without clear correlation with the clinical symptoms. All the patients had had markedly elevated ferritin and LDH levels at the time of diagnosis.

Blood samples for plasma zinc, plasma copper, and blood manganese measurements were collected in heparin‐containing tubes. Plasma zinc concentration was measured by photometry and plasma copper concentration by spectrophotometry. Blood manganese, 24‐h urine zinc and urine copper were measured by inductively coupled plasma mass spectrometry (ICP‐MS; Laboratory: Synlab/MVZ Labor Dr. Limbach & Kollegen, Heidelberg, Germany).

Blood samples for plasma calprotectin measurement were collected in EDTA tubes and they were centrifuged at 3000 rpm for 10 min. Plasma samples were stored in aliquots at −75°C until use. Control plasma was collected from five non‐matched adult volunteer donors with no known illnesses.

Plasma calprotectin was measured with an enzyme‐linked immunosorbent assay (Calprotectin ELISA [ALP]) from the CALPROLAB™ (Lysaker, Norway) according to the instructions of the manufacturer. Absorbance was measured at 405 nm using an ELISA plate reader (Hidex Sense). The standard curve was based on measuring six standards in the range 0–500 ng/mL.

Plasma samples were diluted (due to extremely high calprotectin concentrations, see Results) with the Sample Dilution Buffer (from CALPROLAB) at final dilution 1:10 000. The control samples were analyzed with dilution 1:20.

Samples, standards, and controls were incubated in the duplicate microtiter wells 45 min on a horizontal plate shaker (600 rpm) at room temperature. At the end of the incubation time, the liquid was removed, and the wells were washed three times with Washing Solution (from CALPROLAB), using an automatic plate washer. After washes, the Enzyme Conjugate was added to each well, and the plate was incubated for 45 min on a horizontal plate shaker (600 rpm) at room temperature. After the incubation, the plate was washed as described above. Then Enzyme Substrate Solution was added to each well and the plate was incubated for 25 min at room temperature, protected from light. After the incubation, 100 μL 1 M NaOH was added to each well to stop the reaction.

The data were analyzed using IBM SPSS Statistics 27 software. Correlations were calculated with the Spearman correlation coefficient. *p*‐values < 0.05 were considered statistically significant. This study was approved by the joint Ethics Committee of the University of Turku and Turku University Hospital.

## 3 RESULTS

Characteristics of 10 patients with LPI are presented in Table 1. Plasma zinc concentration was elevated in two patients (median 14.9 μmol/L, range 9–25.3 μmol/L). A 24‐h urine collection was performed for six patients. Only one patient had elevated urinary zinc, otherwise urinary zinc excretion was within the normal range. Plasma copper concentration was within normal range, but urine copper was slightly elevated in three out of six patients. Blood manganese levels were elevated in three patients (median: 10.2 μg/L, range: 1.6–22.6 μg/L). Plasma iron levels were low or normal (median: 11.5 μmol/L, range: 8–20 μmoL/L). Plasma calcium levels were normal in all patients (median: 2.31 mmol/L, range: 2.19–2.45 mmol/L).

**TABLE 1:** Clinical and laboratory data of 10 adult patients with LPI.

	Median (range)	Reference range	
Age	46.5 (27–65)		Years
Sex (M/F)	4/6		
Creatinine	128.5 (76–308)	F: 50–90	μmol/L
		M: 60–100	
Cystatin C	1.46 (0.72–2.31)	0.62–1.11	mg/L
eGFR	50 (17–88)		mL/min/1.73 m^2^
U‐beta‐2 microglobulin	45.2 (0.07–98.9)	0–0.3	mg/L
Hemoglobin	121 (99–138)	117–155	g/L
Leukocytes	4.25 (3.5–6.7)	3.4–8.2	10^9^/L
Platelets	138 (92–248)	150–360	10^9^/L
ALT	28.5 (15–83)	<35	U/mL
ALP	84 (38–173)	35–105	U/L
NH_4_ ion	27 (20–66)	<50	μmol/L
Triglycerides	2.85 (1.4–6.2)	0.45–2.6	mmol/L
LDL	2.9 (1.7–4.3)	<3	mmol/L
Prealbumin	0.28 (0.23–0.38)	0.2–0.4	g/L
Urine lysine	578 (242–855)	2–63	μmol/mmol Krea
Plasma lysine	118 (98–139)	114–289	μmol/L
Plasma arginine	36 (28–63)	15–183	μmol/L
Plasma ornithine	18 (16–34)	22–115	μmol/L
Plasma citrulline	90 (49–146)	0–53	μmol/L
Plasma glutamine	824 (653–1313)	324–781	μmol/L
Plasma zinc	14.9 (12–25.3)	9–18	μmol/L
Plasma copper	16.5 (10.4–25.1)	10.7–26.6	μmol/L
Blood manganese	10.2 (1.6–22.6)	6–11	μg/L
Plasma calcium	2.31 (2.19–2.45)	2.15–2.51	mmol/L
Plasma iron	11.5 (8–20)	9–34	μmol/L
Urine zinc (24 h)	4.2 (1.4–20)	2.3–12	μmol/24 h
Urine copper (24 h)	0.98 (0.25–1.61)	<0.94	μmol/24 h
		Controls	
Plasma calprotectin	622 338 (18 817–1 063 291)	291–1695	μg/L

ALT was elevated in two patients (median: 28.5 U/mL, range: 15–83 U/mL) and ALP was elevated in four patients (median: 84 U/L, range: 38–173 U/L). NH_4_ median was 27 μmoL/L (range: 20–66 μmol/L). Plasma prealbumin levels were normal in all patients.

Seven patients had elevated plasma creatinine (median: 128.5 μmol/L, range 76–308 μmol/L) and serum cystatin C (median: 1.46 mg/L, range: 0.72–2.31 mg/L) levels. GFR was decreased in all patients (median: 50 mL/min/1.73 m^2^, range: 17–88 mL/min/1.73 m^2^). Urine beta‐2 microglobulin was measured from eight patients and was elevated in seven of them (median: 45.2 mg/L, range: 0.07–98.9 mg/L). Six patients had hypertriglyceridemia and three patients had elevated LDL cholesterol.

**FIGURE 1:**
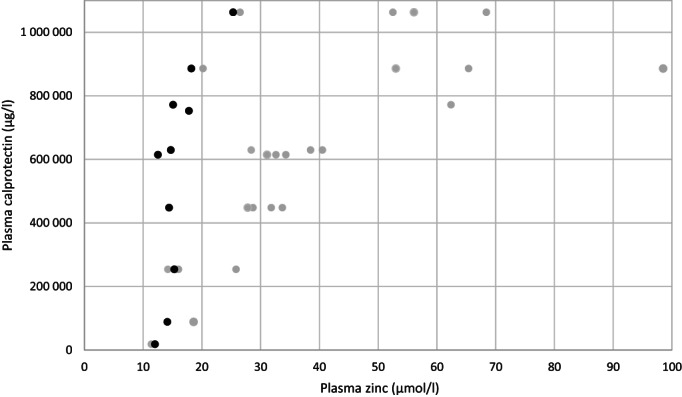
Correlation of plasma calprotectin with plasma zinc in 10 adult LPI patients. Black dots: plasma zinc at the time of the study. Grey dots: plasma zinc in 2005–2012.

Calprotectin was measured from 10 patients using an enzyme‐linked immunosorbent assay (ELISA). In all patients, plasma calprotectin was extremely high (median: 622 338 μg/L, range: 18 817–1 063 291 μg/L) compared to the health controls (median: 608 μg/L, range: 291–1695 μg/L). There was a significant positive correlation between plasma zinc concentration and plasma calprotectin concentration (*r* = 0.806, *p* = 0.005; Figure 1). There was no correlation between renal function markers (GFR, serum cystatin C, and urine beta‐2 microglobulin) and plasma calprotectin levels. Both ALT and ALP correlated with plasma calprotectin (*r* = 0.760, *p* = 0.011, and *r* = 0.721, *p* = 0.019).

## 4 DISCUSSION

We observed extremely high plasma calprotectin concentration in all 10 Finnish LPI patients in our study. It was nearly 1000 times higher in LPI patients than in healthy controls. Elevated extracellular calprotectin levels have been described in several diseases. Generally, plasma calprotectin levels observed in different inflammatory diseases have been much lower than reported in the present study; for example, in rheumatoid arthritis, plasma calprotectin has been less than 20 000 μg/L.^29^ On the other hand, in patients with PAMI syndrome (PSTPIP1‐associated myeloid‐related proteinemia inflammatory syndrome, earlier known as hyperzincemia/hypercalprotectinemia) plasma calprotectin can be even 500–12 000 times the normal levels.^30^, ^31^

Increased serum calprotectin levels have also been reported in patients with active systemic vasculitis and glomerulonephritis.^32^, ^33^ In the glomeruli, infiltrating macrophages produce calprotectin and its subunits. Once released, calprotectin interacts with several receptors, including Toll‐like receptor 4 (TLR4), activating productions of proinflammatory cytokines (e.g., IL‐6, CXCL1, TNF‐α).^34^, ^35^, ^36^ A correlation between calprotectin levels and severity of different forms of glomerulonephritis has, indeed, been reported.^32^ Moreover, in renal biopsies of patients with antineutrophil cytoplasm antibody (ANCA)‐associated vasculitis (AAV), a glomerular infiltration of calprotectin in active crescents as well as areas of focal necrosis have been found. In the patients with acute AAV, serum calprotectin varied from 5000 to 40 000 μg/L.^33^ In addition, Malícková et al. (2010) have reported significantly elevated plasma calprotectin levels in patient with end‐stage renal disease (mean 24 380 μg/L).^37^ It has also been shown that urinary calprotectin is a good diagnostic test for discrimination of intrinsic and prerenal acute renal injury. Unfortunately, we have no data of urinary calprotectin levels in Finnish LPI patients.^38^

Renal insufficiency is a common complication in LPI. In Finland, almost every adult LPI patient has impaired renal function.^39^ Pathogenesis of this complication is still poorly understood although several explanations have been suggested. High concentrations of cationic amino acids are nephrotoxic in animals, and lysine trapped inside the proximal tubular cells is directly nephrotoxic.^40^, ^41^ In kidneys, intracellular arginine synthesis from citrulline may increase due to oral citrulline supplementation, promoting production of nitric oxide and causing damage and apoptosis in glomerular, mesangial, and tubular cells.^42^, ^43^, ^44^ In this study, we measured remarkably high plasma calprotectin concentrations in LPI patients, and it is possible that calprotectin plays a role in the pathogenesis of renal disease. However, the mechanism of increased plasma calprotectin levels in LPI is unclear and more investigation is needed.

Elevated plasma zinc concentrations have been described in patients with LPI. In 2007–2012, plasma zinc was elevated in most of our LPI patients (Table 2), which triggered this study. Surprisingly, in the current study, plasma zinc levels of the same patients were normal or only mildly elevated. However, at the time of the current study, zinc analyses were performed in different laboratory with a different measurement method: in 2007–2012, plasma zinc was measured by atomic absorption spectrometry (AAS) and in 2020–2021 by photometry. This might have had an effect on the results. Furthermore, patients with extremely high plasma calprotectin levels might actually suffer from zinc deficiency since calprotectin has a high binding capacity for zinc.

**TABLE 2:** Plasma zinc concentration in LPI patients in 2005–2012 and at the time of the study 2020–2021.

	Plasma zinc (μmol/L)^a^ in								
Patient number	2005	2006	2007	2008	2009	2010	2011	2012	2020–2021
1	14.2			25.8		35.4		16.0	15.3
2		18.6				25.8			14.1
3		98.5	65.4		53.0	69.8		20.2	18.2
4	11.4					10.3			12.0
5	28.4		40.5	38.5					14.7
6			33.7	31.8	27.8	31.1		28.7	14.4
7			62.4			50.5			15.1
8			52.5	68.4	56.1	25.9	26.5		25.3
9									17.8
10			34.3	32.6	31.1	36.9			12.5

*Note*: Method/Laboratory 2005–2011: AAS/Tykslab. Method/Laboratory 2012: AAS/Yhtyneet Medix laboratoriot Oy. Method/Laboratory 2020–2021: Photometry/ Synlab (subcontractor)/ MVZ Labor Dr. Limbach & Kollegen.

^a^ Reference range was 10–20 μmol/L in 2005–2012 and 9–18 μmol/L in 2020–2021.

We conclude that LPI is a multisystem disease that influences many organ systems and also widely the human metabolism. LPI patients have several clinical and laboratory findings, including growth failure, renal insufficiency, coagulation and immunological abnormalities, and risk of alveolar proteinosis. The mechanisms of many LPI complications are poorly understood. Extremely high plasma calprotectin is a new addition to the peculiarities observed in LPI. This novel clinical finding might suggest previously unknown molecular mechanisms behind the pathogenesis of this multiorgan disease. However, further studies are needed to confirm the relevance of this finding and its significance in the course of LPI as well as its predictive value for onset of renal or systemic complications.

## AUTHOR CONTRIBUTIONS

Mari Kärki, Harri Niinikoski, Laura Tanner, and Tero Soukka were responsible for the study concept and design. Mari Kärki, Harri Niinikoski, Tero Soukka, and Satu Lahtinen were responsible for the acquisition of data. Mari Kärki was responsible for database handling and updating and for statistical analysis. Mari Kärki, Harri Niinikoski, Laura Tanner, Satu Lahtinen, and Tero Soukka were responsible for drafting the manuscript. Harri Niinikoski, Laura Tanner, and Tero Soukka were responsible for critical revision of the manuscript for important intellectual content. Harri Niinikoski is the Guarantor for the article.

## FUNDING INFORMATION

This research received no specific grant from any funding agency in the public, commercial, or not‐for‐profit sectors.

## CONFLICT OF INTEREST STATEMENT

The authors declare no conflicts of interest..

## ETHICS STATEMENT

All procedures followed were in accordance with the ethical standards of the responsible committee on human experimentation (institutional and national) and with the Helsinki Declaration of 1975, as revised in 2000(5). Informed consent was obtained from all patients for being included in the study.

## Supporting information


**Supplemental Table 1.** Individual plasma creatinine, plasma cystatine C, plasma calcium, plasma zinc, urine zinc (24 h) and plasma calprotectin values.

## Data Availability

The data that support the findings of this study are available in the supplementary material of this article.
