# Supplementary material for: Plasma calprotectin is extremely high in patients with lysinuric protein intolerance
Source: JIMD Rep. 2023 Jun 20;64(4):293–9. doi: 10.1002/jmd2.12377 (PMC10315390; doi:10.1002/jmd2.12377)
Supplement: Supplementary file 1 — Supplemental Table 1. Individual plasma creatinine, plasma cystatine C, plasma calcium, plasma zinc, urine zinc (24 h) and plasma calprotectin values. [file JMD2-64-293-s001.docx]

| **Supplemental Table 1** Individual plasma creatinine, plasma cystatine C, plasma calsium, plasma zinc, urine zinc (24h) and plasma calprotectin values. | | | | | | | | | | | |
| --- | --- | --- | --- | --- | --- | --- | --- | --- | --- | --- | --- |
|  | Reference range | 1 | 2 | 3 | 4 | 5 | 6 | 7 | 8 | 9 | 10 |
| Sex |  | f | f | f | f | m | f | m | f | m | m |
| Creatinine | f: 50-90 µmol/l | 90 | 76 | 308 | 160 | 83 | 192 | 122 | 135 | 191 | 104 |
|  | m: 60-100 µmol/l |  |  |  |  |  |  |  |  |  |  |
| eGFR | > 90 ml/min/1.73 m² | 67 | 88 | 17 | 32 | 85 | 25 | 60 | 39 | 40 | 74 |
| Cystatin C | 0.62-1.11 mg/l | 0.96 | 0.72 | 2.31 | 2.00 | 1.13 | 1.89 | 1.52 | 1.40 | 1.60 | 1.06 |
| Plasma calcium | 2.15-2.51 mmol/l | 2.20 | 2.29 | 2.32 | 2.19 | 2.34 | 2.45 | 2.37 | 2.3 | 2.27 | 2.37 |
|  |  |  |  |  |  |  |  |  |  |  |  |
| Plasma zinc | 9-18 µmol/l | 15.3 | 14.1 | 18.2 | 12 | 14.7 | 14.4 | 15.1 | 25.3 | 17.8 | 12.5 |
| Urine zinc (24h) | 2.3-12 µmol/24h |  |  | <5.4 |  |  | 1.4 | 4.2 | 3.0 | 8.6 | 20.0 |
| Plasma Calprotectin | Controls: 291-1695 µg/l | 254481 | 88618 | 886435 | 18817 | 629793 | 448148 | 772277 | 1063291 | 752983 | 614882 |
|  |  |  |  |  |  |  |  |  |  |  |  |
| f, female; m, male |  |  |  |  |  |  |  |  |  |  |  |
